# Atypical audio-visual neural synchrony and speech processing in early autism

**DOI:** 10.1186/s11689-025-09593-w

**Published:** 2025-02-18

**Authors:** Xiaoyue Wang, Sophie Bouton, Nada Kojovic, Anne-Lise Giraud, Marie Schaer

**Affiliations:** 1 Auditory Language Group, Department of Basic Neuroscience, University of Geneva, Geneva, Switzerland; 2 Institut Pasteur, Université Paris Cité, Hearing Institute, Paris, France; 3 Autism Brain & Behavior Lab, Department of Psychiatry, University of Geneva, Geneva, Switzerland; 4 Department of Medical Psychology and Ethics, School of Basic Medical Sciences, Cheeloo College of Medicine, Shandong University, Jinan, Shandong China

**Keywords:** Autism spectrum disorders (ASD), Gaze direction, Speech envelope, Visual motion, Audio-visual, Oscillation phase entrainment

## Abstract

**Background:**

Children with Autism Spectrum disorder (ASD) often exhibit communication difficulties that may stem from basic auditory temporal integration impairment but also be aggravated by an audio-visual integration deficit, resulting in a lack of interest in face-to-face communication. This study addresses whether speech processing anomalies in young autistic children (mean age 3.09-year-old) are associated with alterations of audio-visual temporal integration.

**Methods:**

We used high-density electroencephalography (HD-EEG) and eye tracking to record brain activity and gaze patterns in 31 children with ASD (6 females) and 33 typically developing (TD) children (11 females), while they watched cartoon videos. Neural responses to temporal audio-visual stimuli were analyzed using Temporal Response Functions model and phase analyses for audiovisual temporal coordination.

**Results:**

The reconstructability of speech signals from auditory responses was reduced in children with ASD compared to TD, but despite more restricted gaze patterns in ASD it was similar for visual responses in both groups. Speech reception was most strongly affected when visual speech information was also present, an interference that was not seen in TD children. These differences were associated with a broader phase angle distribution (exceeding pi/2) in the EEG theta range in children with ASD, signaling reduced reliability of audio-visual temporal alignment.

**Conclusion:**

These findings show that speech processing anomalies in ASD do not stand alone and that they are associated already at a very early development stage with audio-visual imbalance with poor auditory response encoding and disrupted audio-visual temporal coordination.

**Supplementary Information:**

The online version contains supplementary material available at 10.1186/s11689-025-09593-w.

## Background

Newborns are immediately attracted to the human voice. In-utero exposure to speech sounds enables them to accurately discriminate speech sounds at birth [1–3]. Since vision develops with a delay relative to hearing, babies only progressively discover that vocal stimuli are related to facial movements. Unlike typically developing (TD) children, children with ASD do not show this primary interest in speech [4–7]. Instead, they tend to engage in slow and repetitive visual exploration of their environment, eventually leading to atypical interests [8–13]. This focus on visual aspects of their surroundings allows ASD children to explore the world at their own pace and avoid highly dynamic stimuli such as speech and biological motion [14–16], which are often perceived as overwhelming [17, 18].

A basic auditory dysfunction in ASD might lead to speech-processing anomalies that in turn cascade into a decreased interest in speech [19–24]. Atypical speech processing becomes apparent early in development, and the fact that the associated neural anomalies in delta, theta, and gamma oscillations, accurately predict the severity of future language deficits suggests that they are causal to difficulties in language comprehension and production [25–28]. The tendency of children with ASD to prefer static or slow visual processing [29–32] possibly exacerbates speech reception challenges by counteracting dynamic audio-visual interaction, a crucial process for speech reception in ecological (e.g., noisy) environments [33–36]. Accordingly, excessively long integration time windows for audio and visual stimuli have been reported in children with ASD [31, 32], implying disturbed audio-visual integration.

Two essential mechanisms participate in audio-visual integration. The first one is the relative timing of auditory and visual stimuli: when these stimuli fall about 250 ms apart, they are often perceived as a single event, potentially influencing each other (e.g. the McGurk effect [37, 38]). The second mechanism is the re-synchronization provoked by the stimulus in one sensory modality on the neural responses to the other one [39–44]. Orofacial visual movements typically precede speech onset and lead to an auditory re-synchronization that sharpens responses to speech [39, 45]. Independent from the integration/fusion of the exact visual and speech content, visual re-synchronization enhances speech processing by boosting the tracking of the speech’s syllabic structure. While anomalies of audio-visual (AV) binding (AV vs. A + V in ERP study) which tranditionally studied over short time windows in ASD are well documented [31, 32, 46–48], how audio and visual signals dynamically synchronize, involving the rhythmic synchronization of auditory and visual signals over longer periods, such as during natural speech exchanges, or when watching movies, remain hypothetical. Dynamic synchronization reflects the capacity for rapid re-synchronization with zero lag across an extended time course.

Auditory and visual sensory processing both operate rhythmically [49–53]. Visual speech information is characterized by a dominant 2–7 Hz rhythm (theta band [54]) and these quasiperiodic visual cues influence speech perception by modulating auditory neuronal oscillations within the same theta range at about 5 Hz [55–60], corresponding to the typical AV integration temporal time around 250ms [39, 61–64]. The resetting of auditory neural oscillations triggered by visual input [65] rhythmically enhances auditory processing [66], a phenomenon that is already observable in typical children [67]. Despite the documented presence of auditory processing anomalies in ASD at around 3 years [24], we still do not know whether they are associated with dynamic audio-visual synchrony anomalies.

This study aims to fill this gap by investigating with high-density EEG the dynamics of auditory and visual processing in young children with and without ASD, aged 1.13 to 5.56 years old, under naturalistic audio-visual conditions, i.e., when children are watching a popular cartoon adapted to their age. The goal is to compare the quality of the neural encoding/decoding of dynamic auditory and visual stimuli and audio-visual temporal coordination across groups.

## Methods

### Participants

Participants were selected from the Geneva Autism Cohort, a longitudinal study that aims to better understand the developmental trajectories in young children with ASD. This cohort’s protocol has been detailed in previous studies [24, 68, 69]. In this study, we used clinical and behavioral assessments, as well as the electroencephalogram (EEG) recorded simultaneously with eye-tracking while children were watching popular cartoon videos.

The sample comprised 31 children with ASD (6 females, mean age = 3.09 years, SD = 0.91, age range: 1.74–5.14) and 32 TD peers (11 females, mean age = 2.95 years, SD = 1.31, age range: 1.31–5.56). Selection criteria for all participants included: age below 6 years, data collected during the participant’s initial visit (i.e. at autism diagnosis for the autistic group), clear and accurate markers associated with movie onset, usable raw data for four different movies, and focus on the screen throughout all recordings. The age difference between the two groups was not significant (Bayesian independent samples t-test, BF10 = 0.287).

Autism clinical diagnoses were meticulously confirmed using standardized tools: either the Autism Diagnosis Observation Schedule-Generic (ADOS-G) [70] or the Autism Diagnosis Observation Schedule, Second Edition (ADOS-2) [71]. Recruitment of participants occurred through specialized clinical centers and community-wide announcements. For the TD group, exclusion criteria included any suspicion of atypical psychomotor development, a history of neurological or psychological disorder, or having a first-degree relative with an autism diagnosis. Table 1 summarizes the clinical characteristics of the ASD and TD samples.

**Table 1:** Participants’ demographic information and group comparison of behavioral tests

			Group					
			ASD (*N* = 31, 6 females)		TD (*N* = 32, 11 females)		Bayesian independent samples t-test	
			Mean	SD	Mean	SD	BF10	error%
Age(in years)			3.092	0.913	2.947	1.308	0.287	0.011
		Range	1.74–5.154		1.31–5.56			
ADOS			7.742	1.879	1.031	0.177	1.283E25	4.105E-28
		Range	4–10		1–2			

### Stimuli and procedure

**Fig. 1:**
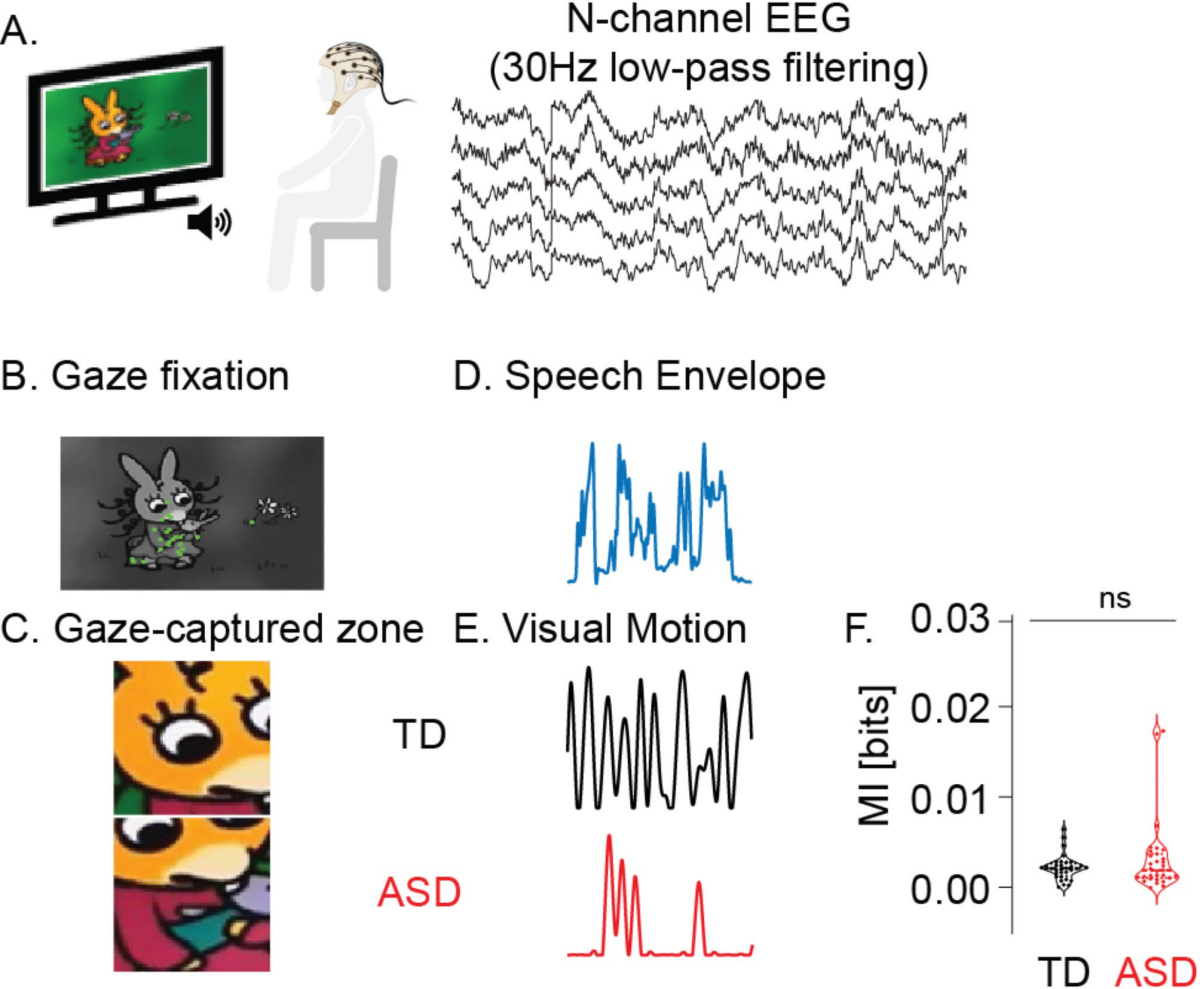
Overview of Experimental Procedures and Features of Interest. **A** Experimental procedures **B** Gaze fixation. Example of individual gaze fixation points (green dots) on a black and white image; **C** Example of gaze-captured screen areas. Depiction of screen areas captured by the gaze of participants in ASD and TD (Typically Developing) groups. **D** Example of a stimulus speech envelope from the cartoon soundtrack. **E** Visual motion corresponds to the same stimulus in each group. **F** Comparison of speech envelope and visual motion. Mutual Information (MI) between ASD and TD groups (ns. *p* > 0.05)

To explore cortical processing of audio-visual stimuli, we employed a passive and naturalistic task suitable for young children. This task involved viewing an age-appropriate French cartoon “TROTRO” [72–75] (example: https://www.youtube.com/watch?v=jT9C9WCIQr8%26t=81s). The selection of “TROTRO” was based on its cognitive accessibility and appeal to the target age group, including kids with ASD. The main character’s verbal interactions enabled isolation of speech and visual motion for brain response analysis. Participants watched four 2.5-minute episodes in a consistent order. Visual engagement was monitored using Tobii Studio, an embedded application of Tobii TX300 eye tracking system. The videos were displayed on a screen with dimensions of 1200 pixels in height (29°38’ visual angle) and 1920 pixels in width (45°53’) with a refresh rate of 60 Hz, optimized for children’s viewing comfort, with participants seated approximately 60 cm from the screen (Fig. 1A).

#### Eye-tracking acquisition and analysis

Gaze data were collected using the Tobii TX300 eye-tracking system (https://www.tobiipro.com), which operates at a sampling rate of 300 Hz. The cartoon was displayed in a frame that provided a visual angle of 26°47’(height) × 45°53’(width). Calibration was performed using a child-friendly procedure integrated into the Tobii system. To maintain consistency and reliability in data quality, we ensured constant lighting conditions in the testing room throughout all sessions. Special consideration was given to the youngest participants, who were seated on their parent’s lap when they felt it more comfortable, a strategy that effectively minimized head and body movements that could have interfered with accurate data collection. We used the Tobii IV-T Fixation filter [76] to extract fixation data, offering precise measures of visual attention and gaze patterns. Inspired by previous findings of altered gaze distribution in autistic children [13, 69], this study used retinal stimuli around the gaze-fixation point as the source of visual information for subsequent analyses.

#### Audio and visual stimuli

We edited the movie soundtrack using Audacity v.2.2.1 to isolate speech excerpts, removing background noise like birdsong and music. Speech envelope data were extracted using the absolute value of the analytic signal [77], downsampled to 1000 Hz, and filtered with a 40 Hz zero-phase Butterworth filter. Visual motion data were tied to participants’ gaze, focusing on stimuli within an 8-degree diameter [78, 79] around the retinal fixation point (318 × 318 pixels) (Fig. 1A1 & A2). The region was converted to grayscale, and luminance differences between successive frames exceeding a threshold of 10 were averaged to represent visual motion [80]. Visual motion was upsampled to match the EEG sampling rate (1000 Hz). Speech envelopes and visual motion were aligned, providing individualized stimulus data based on participants’ gaze patterns for further analysis.

#### Stimulus features analysis

In order to assess shared information between speech envelope and visual motion, in autism and TD groups, we calculated mutual information (MI) scores, a dynamic metric, expressed in bits, which quantifies the reduction in uncertainty of one variable when another is observed [77, 78]. We calculated MI using the *quickMI* function from the Neuroscience Information Theory Toolbox [79]. The parameters for this calculation were set to 4 bins, no delay, and a p-value threshold of 0.001 [79]. For generating the MI scores, we concatenated all kept excerpts in the same sequence across subjects, separately for each stimulus feature and each group (ASD and TD). This process was followed by a comparative analysis of MI values between groups.

We only included stimuli corresponding to time periods with usable EEG signals. This resulted in slight variations in the stimulus duration for the ASD and TD groups, which were controlled for. No significant disparities in MI scores emerged between the two groups (*t*(1, 61) = 1.250, *p* = 0.216, Cohen’s d = 0.315, Fig. 1C), indicating that these minor differences did not lead to notable group differences in the shared information between the speech envelope and visual motion.

### EEG acquisition and pre-processing

The EEG data were acquired using a 129-electrode (Hydrocel Geodesic Sensor Net (HCGSN) system (Electrical Geodesics, USA) at a sampling rate of 1000 Hz. During recording, the signals were subjected to real-time 0–100 Hz band-pass filtering. The reference electrode was positioned at the vertex (Cz). Data pre-processing was conducted using the EEGLAB v2019 toolbox within the MATLAB environment [80] and Cartool (https://sites.google.com/site/cartoolcommunity/). One hundred and ten channels were kept, excluding the cheek and neck electrodes to prevent contamination by muscle artifacts. EEG signals were filtered using a zero-phase fourth-order Butterworth bandpass (0.1–70 Hz) and a 50 Hz notch filter to eliminate power line noise. EEG data were visually inspected to remove movement artifact-contaminated periods. Bad channels were identified and excluded for exhibiting excessive signal amplitude. Eye blinks, saccades, electrical noise, and heartbeat artifacts were removed using independent component analysis (ICA). A spherical spline interpolation was used to interpolate the channels contaminated by noise using the ICA-corrected data. Finally, a common average reference was recalculated on the cleaned data, with an additional step of applying a 30 Hz low-pass filtering [81]. To ensure that all the EEG signals and stimulus features were on a similar scale and thus comparable, we normalized both the EEG signals and stimulus features (i.e. speech envelope and visual motion) using the *nt_normcol* function (Noisetools: http://audition.ens.fr/adc/NoiseTools/*).*

### Temporal response functions (TRF)

To quantify how well EEG in ASD and TD children linearly varied with the stimulus features, we performed regularized regression (with ridge parameter λ) as implemented in the mTRF toolbox [82]. The TRF models the strength and direction of the brain’s response to stimulus features, such as speech envelope or visual motion, at specific time lags. For the TRF modeling, we downsampled all signals to a rate of 100 Hz to accelerate computation.

#### Estimation of TRF using forward encoding models

We used a forward encoding model to predict EEG responses over time lags from 300 ms before to 300 ms after the stimulus. Separate univariate models were constructed for auditory (speech envelope, A-only) and visual (visual motion, V-only) stimuli. Additionally, a multivariate model (AV-joint) integrated both regressors, using trade-off weights to balance auditory and visual contributions, ensuring balanced representation of multisensory integration.

We compared the AV-joint model with A-only and V-only models to assess the benefits of audio-visual integration over unimodal processing. Using an n-fold leave-one-out cross-validation strategy, “generic” models were created to predict individual EEG data from TRFs derived from other participants. Model performance was optimized through a parameter search for the regularization parameter λ (see supplementary methods for λ selection). Pearson’s correlation coefficient (r) between EEG signals with TRF-predicted signals was used to quantify model prediction accuracy per electrode. Then, correlations were averaged across participants to create a scalp-wide map of prediction accuracy. Finally, prediction accuracy was converted into Z-scores for statistical comparison by subtracting the surrogate data mean and dividing by its standard deviation. Surrogate distributions were generated by randomly shifting testing EEG segments, maintaining temporal structure. This analysis evaluated how accurately stimulus features were predicted from EEG data for each participant.

To quantitatively compare the accuracy between the ASD and TD groups, we used a cluster-based permutation test with 1000 randomization iterations, following the approach of Maris and Oostenveld [83]. Clusters were defined by considering both time and spatial electrode configurations, requiring each cluster to include at least two adjacent electrodes. A pivotal aspect of this approach was to ensure that the cluster-level type-I-error probability remained below the 0.05 threshold. This strategy was effective in controlling the family-wise error rate, maintaining it within the 5% type-I-error rate boundary.

#### Stimulus reconstruction using decoding models

We trained EEG decoders within a -300 to 300 ms post stimulus onset temporal window, using leave-one-out cross-validation and optimization to assess the accuracy of stimulus reconstruction (, i.e., speech envelope and visual motion). This approach allows us to identify the most informative segments for decoding, i.e. the time-lags with the highest EEG-stimulus synchronization. To determine the accuracy of stimulus reconstruction and select the best time lags, we used the Kruskal-Wallis test with Dunn’s multiple comparisons test [84]. This non-parametric statistical method was chosen for its capability to handle variations in group means and variances across different conditions.

### Low-frequency tracking of audio-visual signal

To explore whether the combined processing of auditory and visual stimuli relies on the tracking of audio-visual signals by low-frequency brain activity, we used a coherence-based and phase-based analytical framework [85]. This approach probed the interplay between neural responses and stimulus features by comparing their magnitude spectra and the phase relationship. Our analyses are centered on the delta and theta frequency bands, which are critical for effective integration of multimodal information [86].

#### Coherence analysis

We assessed individual responses to speech envelope and visual motion by computing magnitude-squared coherence for each trial and electrode using the *mscohere* function in Matlab, applying Welch’s averaged modified periodogram method. The analysis spanned a frequency range from 0.1 to 30 Hz, in 0.33 Hz steps [87].

The analysis targeted delta (δ, ~ 4 Hz) and theta (θ, 4–8 Hz) frequency bands, identifying frequencies where coherence peaked most prominently for each stimulus condition. Statistical comparisons across groups and stimuli were conducted using the clusters identified through the method outlined in Sect. 4, combined with a nonparametric test and Dunn’s multiple comparisons test [84]. Statistical significance was established using a surrogate-corrected coherence approach. Surrogate distributions were generated by randomly shifting the neural time course relative to the stimulus feature time courses, preserving their original temporal structure. This process was repeated 50 times for each stimulus condition to generate a robust surrogate distribution. The resulting coherence values were then standardized (Z-scored) against this distribution.

#### Phase analysis

We also performed a phase analysis by calculating the cross-power spectral density (CPSD) phase for each stimulus, electrode, and trial. This was done using the *cpsd* function in Matlab, employing parameters consistent with the coherence analyses. Phase values were determined based on the peak frequency identified in the coherence analysis. Group comparisons were conducted using Matlab’s Circular Statistics Toolbox [88]. A two-way parametric ANOVA for circular data was performed to facilitate a nuanced comparison between pairs of conditions and groups, followed by post-hoc comparisons using the Watson-Williams multi-sample test [88]. Meanwhile, the Rayleigh test was used to investigate whether phase distribution was unimodal. Our focus was primarily on electrodes identified through TRF estimation outcomes.

## Results

### Atypical speech envelope processing in autistic children

**Fig. 2:**
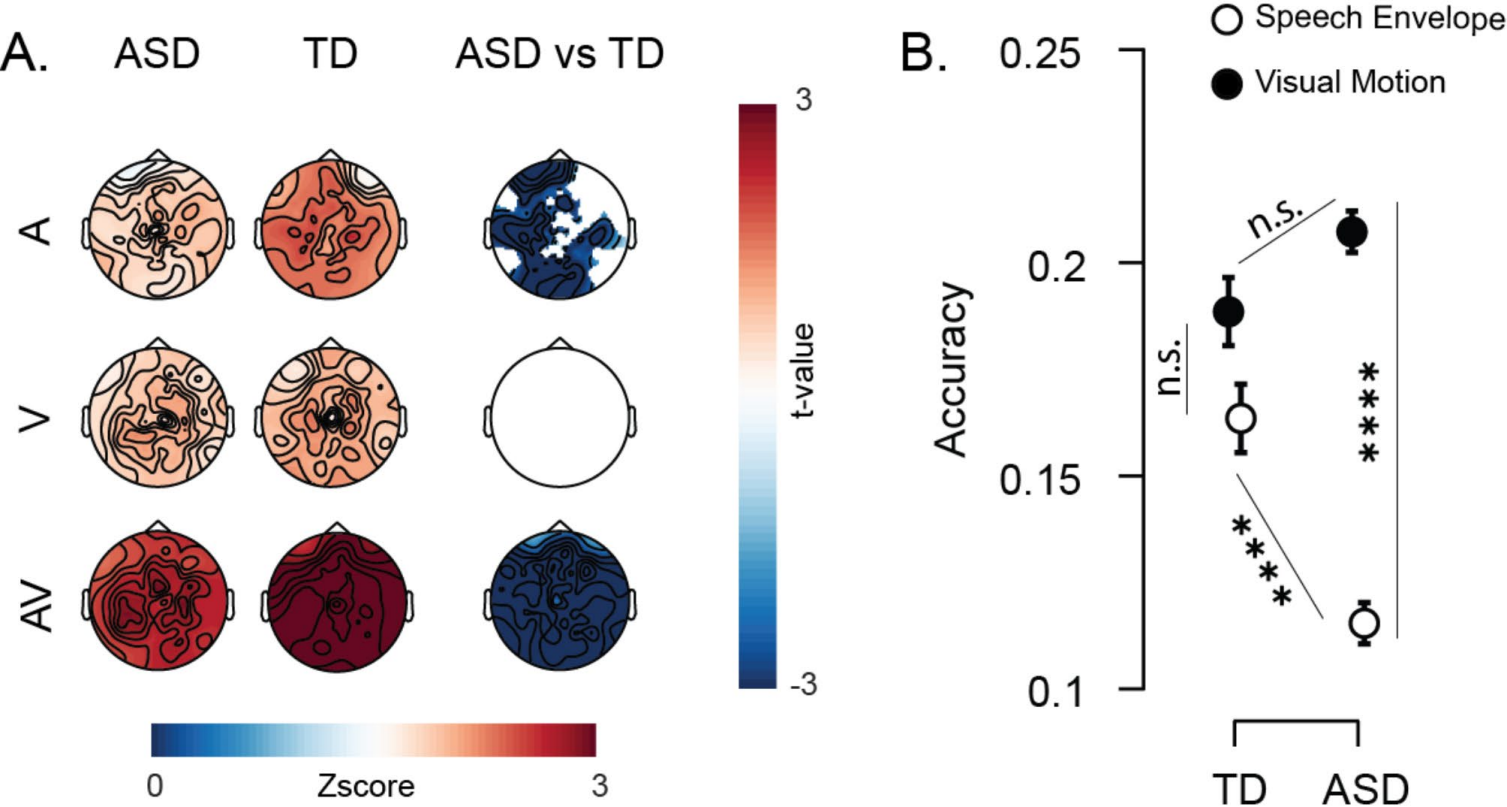
Comparison of audio, visual, and AV models. **A**. Neural representations in ASD (left) and TD (middle) groups, for each model (A-speech envelope, V-visual motion, and AV joint) across all scalp electrodes. The right column shows EEG channels where significant group differences are observed using cluster-based nonparametric statistics (*p* < 0.05; with a positive t-value indicating greater predictability in the ASD group compared to the TD group). **B**. Stimulus reconstruction accuracy for speech envelope and visual motion in both groups. Error bars indicate the standard error of the mean. Significance levels are indicated as follows: ‘ns’ for p > 0.05 (not significant), * for p < 0.05, ** for p < 0.01, *** for p < 0.001, and **** for p < 0.0001

We found distinct neural tracking patterns for the auditory and visual parts of the stimuli. Different scalp distribution patterns were observed between the two groups (Fig. 2) with cluster-correction *p* < 0.05. The ASD group had reduced neural response to the speech envelope relative to the TD group (Fig. 2A, top row). In contrast, there was no between-group difference in visual motion processing (Fig. 2A middle row).

A univariate stimulus reconstruction accuracy measure was compared between the ASD and TD groups, and aligned with the neural tracking findings, suggesting that speech processing is primarily impaired in autistic children. While reconstruction accuracy was comparable for speech envelope and visual motion (*p* > 0.9999, Fig. 2B) in the TD group, it was lower for speech than for visual motion (*p* < 0.0001, Fig. 2B) in the ASD group. These results suggest intact visual processing dynamic communicative stimuli in young children with ASD but atypical auditory processing.

### Audiovisual integration anomaly in autism disrupts visual enhancement of auditory processing

We then explored whether speech anomalies in autism are limited to auditory processing difficulties or associated with audiovisual (AV) processing anomalies. First, the joint model suggested weaker AV representation in the ASD than in the TD group (Fig. 2A bottom row) and we found the expected stronger neural representation of the combined AV stimulus compared to individual single stimuli in both groups.

**Fig. 3:**
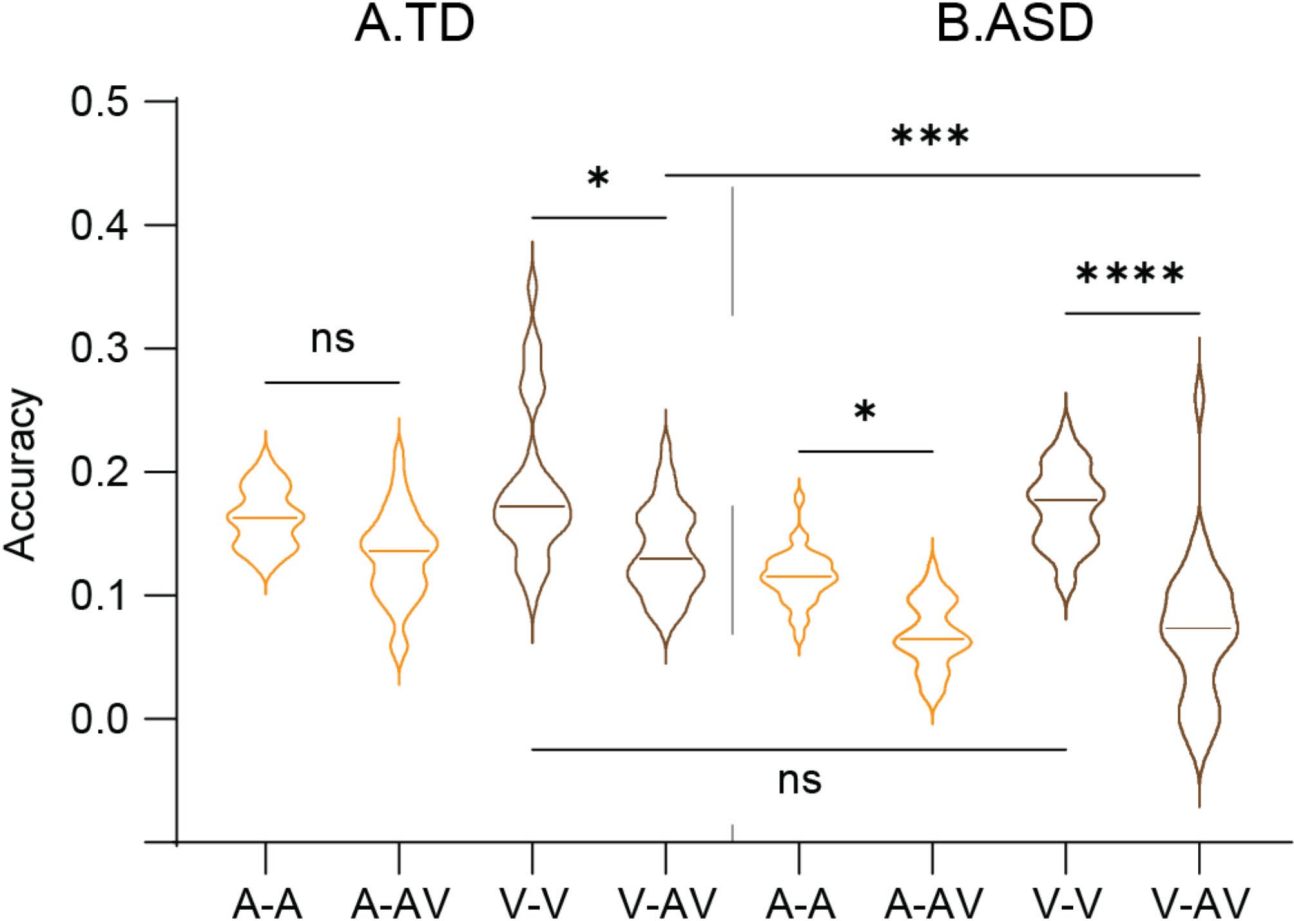
Evaluation of decoding accuracy in TD **A** and ASD **B.** Stimulus reconstruction accuracy: speech envelope(A-) and visual motion(V-) in both the single-stimulus model (A-only = A-A and V-only = V-V) and the AV-joint model (A-AV, and V-AV)). Significance levels are indicated as follows: ‘ns’ for p > 0.05 (not significant), * for p < 0.05, ** for p < 0.01, *** for p < 0.001, **** for p < 0.0001. For additional details, see Supplemental Figs. 3 − 1

Distinct audiovisual integration patterns in ASD and TD children (Table 2; Fig. 3) were found by comparing the decoding accuracies for speech envelope and visual motion across univariate (A-only and V-only) and multivariate (AV-joint) models. Notably, in the TD group, the accuracy of speech envelope reconstruction in the AV-joint model (concurrent speech envelope and visual motion) did not significantly differ from the A-only model (*p* = 0.1414, Fig. 3A). Conversely, in the ASD group, the speech envelope was less accurately decoded in the AV-joint model than in the A-only model (*p* = 0.0304, Fig. 3B), indicating a disruptive effect of AV integration on auditory processing specific to this group. A decrease in visual motion reconstruction accuracy in the AV-joint model compared to the V-only model was observed in both groups (ASD group: *p* < 0.0001, TD group: *p* = 0.0373, Fig. 3), with a more pronounced decrement observed in the ASD group (*p* = 0.0003, Table 3). These findings suggest that integrating AV speech signals has a cost on on visual processing, and that this cost is higher in children with ASD.

**Table 2:** The statistical difference among joint (AV) model and single models (A- &V-) for speech envelope and visual motion

	Dunn’s multiple comparisons test	Mean rank diff.	Significant?	Adjusted *P* Value
ASD	speech envelope	-60.48	Yes	0.0304
	visual motion	-137.9	Yes	< 0.0001
TD	speech envelope	-51.09	No	0.1414
	visual motion	-58.47	Yes	0.0373

**Table 3:** The statistical difference of the decoding accuracy evolution for speech envelope and visual motion

	Dunn’s multiple comparisons test	Mean rank diff.	Significant?	Adjusted *P* Value
Visual motion	ASD v.s.TD	-37.55	Yes	0.0003
Speech envelope	ASD v.s.TD	-13.32	No	0.8866
TD	V vs. A	-6.094	No	> 0.9999
ASD	V vs. A	-30.32	Yes	0.0065

A: speech envelope

V: visual motion

**Fig. 4:**
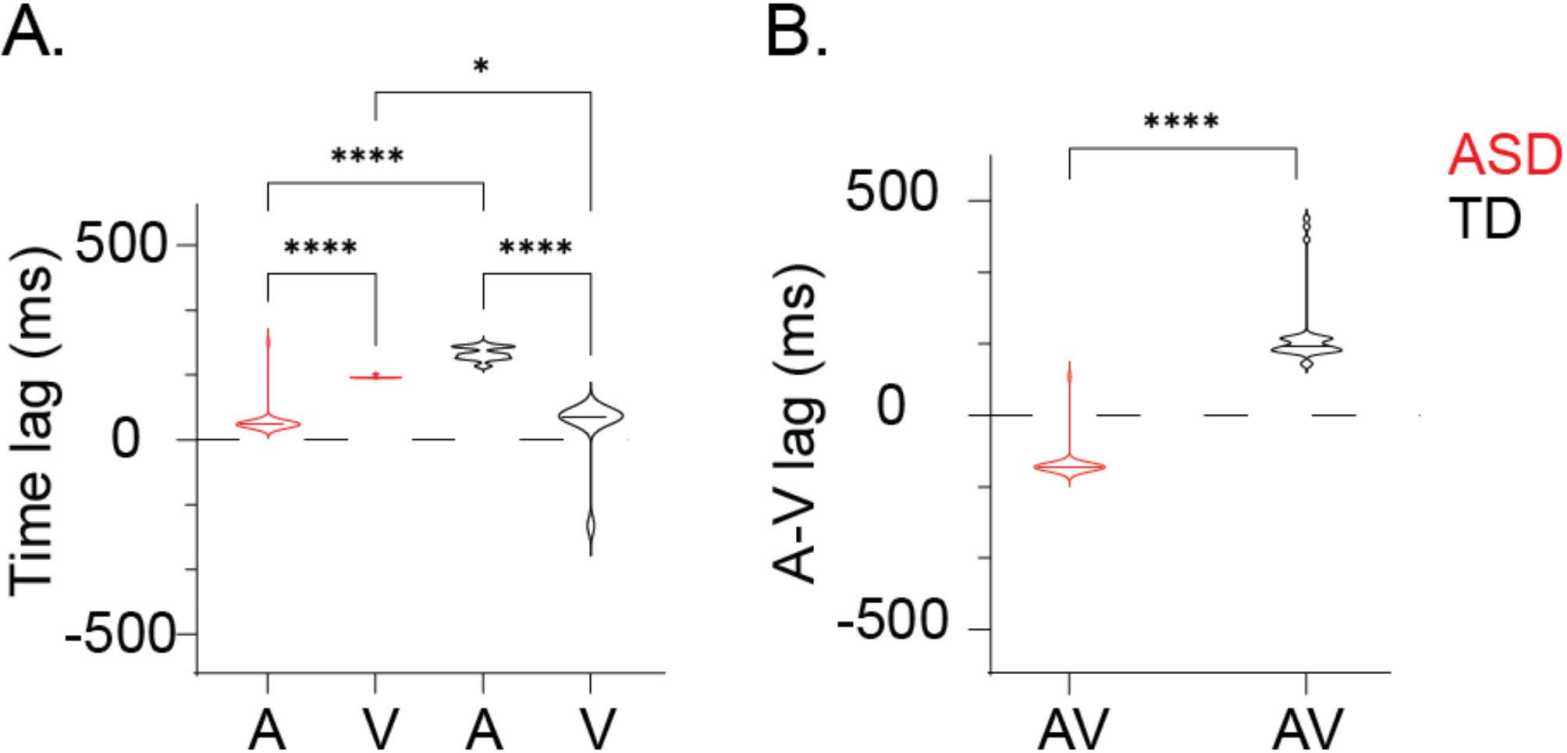
Optimal EEG-stimuli time lag for ASD (red) and TD (black) groups. **A** depicts the optimal time-lag observed in the reconstruction of stimulus features in AV-joint model, specifically speech envelope (A-) and visual motion(V-); Positive values represent stimulus lead EEG signal. **B** illustrates the A-V time lag AV-joint model. Positive values represent V leads A. Significance levels are indicated as follows: ns > 0.05, *p < 0.05, **p < 0.01, ***p < 0.001, ****p < 0.0001

Surprisingly, we found distinct time-lags in auditory and visual decoding accuracies between ASD and TD when analyzing the temporal dynamics of audiovisual integration. In the TD group (Fig. 4A), auditory decoding reached significance at ~ 200 ms, while visual decoding took ~ 50 ms. In contrast, the ASD group showed the opposite pattern: ~200 ms for visual decoding and ~ 50 ms for auditory decoding (Fig. 4B). This reveals a visual lead in TD children, aligning with visual cues typically preceding sounds, but an auditory lead in autistic children, indicating a fundamental shift in sensory processing order.

### Theta-range desynchronization of audio-visual responses in autism

**Fig. 5:**
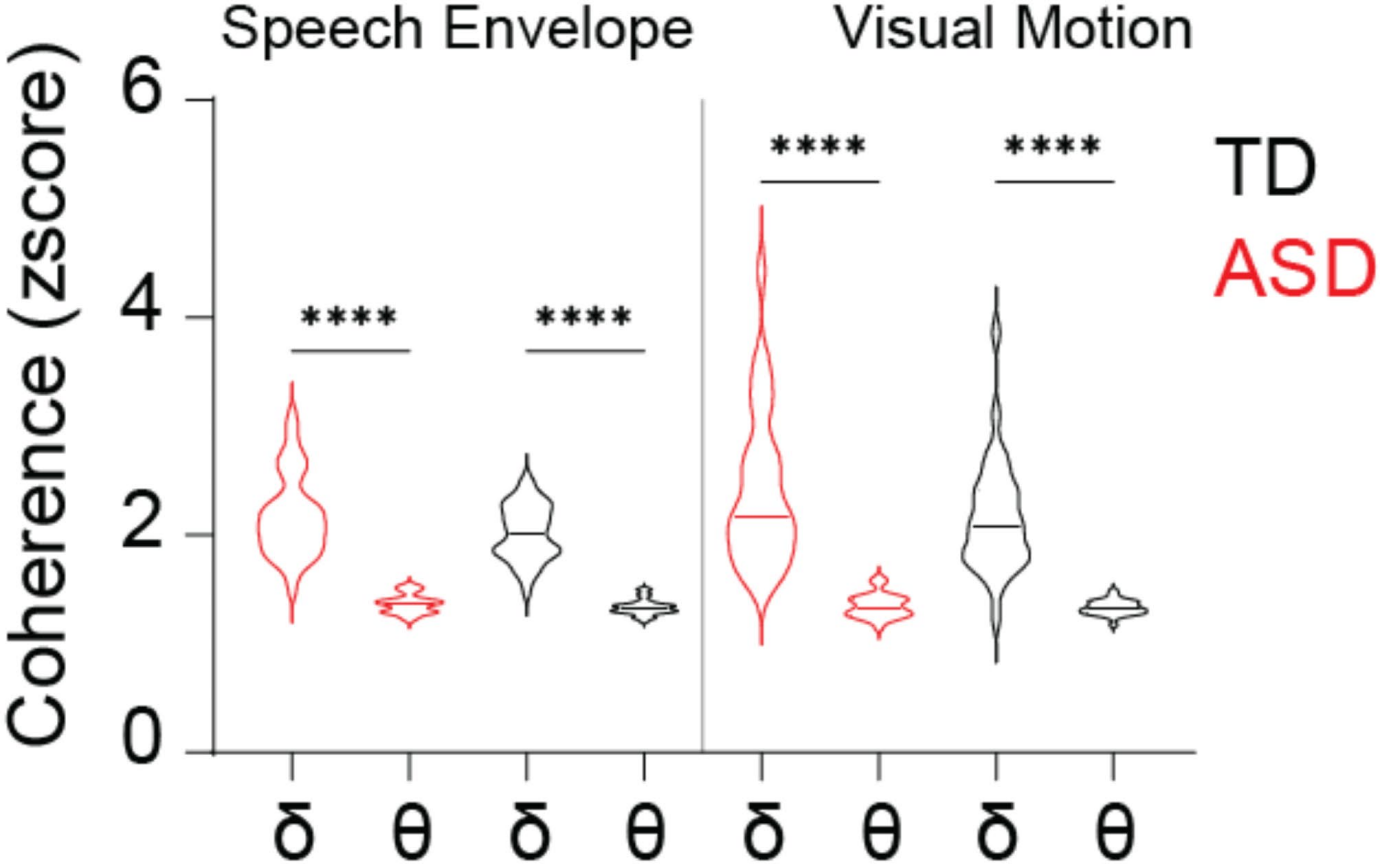
Stimulus-response coherence in Theta and Delta Bands for ASD (red) and TD (black) groups. The plot displays the coherence between stimulus and response for Speech Envelope and Visual Motion. Error bars represent the standard error of the mean. The coherence levels are compared within the specific frequency bands of interest, highlighting potential group differences in sensory processing. Significance levels are indicated as follows: ns > 0.05, *p < 0.05, **p < 0.01, ***p < 0.001, ****p < 0.0001

To determine whether speech tracking anomalies in ASD stem from general stimulus/brain synchronization deficits or audiovisual integration issues, we analyzed the stimulus-response relationships in the delta (1–4 Hz) and theta (4–8 Hz) bands. Both groups showed higher stimulus/brain coherence in the delta than the theta band, with remarkably similar average coherence values and distribution patterns across groups (Fig. 5; Table 4). This indicates that the capacity of neural synchronization to auditory and visual stimuli is consistent in ASD and TD groups.

**Table 4:** The statistical difference across groups and frequency bands in stimulus-response coherence

		Dunn’s multiple comparisons test	Mean rank diff.	Significant?	Adjusted *P* Value
ASD	A	delta vs. theta	162.7	Yes	< 0.0001
ASD	V	delta vs. theta	191.5	Yes	< 0.0001
ASD	delta	A vs. V	-7.774	No	> 0.9999
ASD	theta	A vs. V	20.94	No	> 0.9999
TD	A	delta vs. theta	178.6	Yes	< 0.0001
TD	V	delta vs. theta	176.4	Yes	< 0.0001
TD	delta	A vs. V	-0.6563	No	> 0.9999
TD	theta	A vs. V	-2.844	No	> 0.9999
A	delta	ASD vs. TD	12.3	No	> 0.9999
A	theta	ASD vs. TD	28.19	No	> 0.9999
V	delta	ASD vs. TD	19.42	No	> 0.9999
V	theta	ASD vs. TD	4.406	No	> 0.9999

A: speech envelope

V: visual motion

**Fig. 6:**
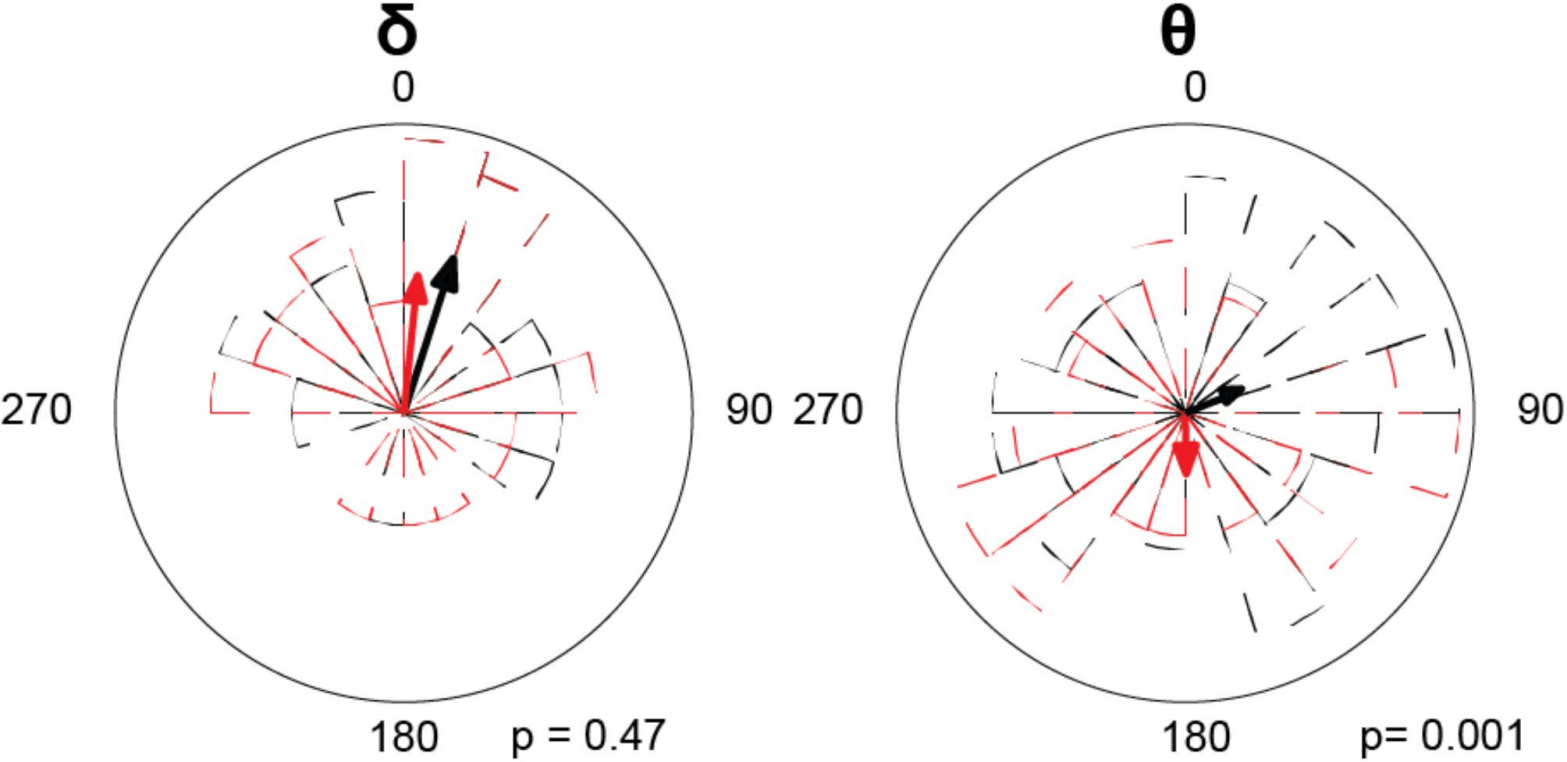
Phase-shift distribution between speech envelope and visual motion. This figure shows the phase-shift distribution between the brain processes of speech envelope and visual motion stimuli for each group. The circular mean of the phase-shift across all subjects is indicated by colored lines: red for the ASD group and black for the TD group. Corresponding polar histograms in red (ASD) and black (TD) visually represent the distribution of phase-shifts for each group. Both groups were tested against the hypothetical uniform distribution of delta (rayleigh test, ASD: *p* < 0.001, rayleigh *r* = 0.98, TD: *p* < 0.001, rayleigh *r* = 0.98) and theta phase (rayleigh test, ASD: *p* < 0.001, rayleigh *r* = 0.95, TD: *p* < 0.001, rayleigh *r* = 0.96)

Given the preserved synchrony between brain activity and external stimuli in each modality, we then sought whether audio-visual integration anomalies, notably the inverted AV temporal patterns, are associated with a phase desynchronization of auditory and visual processing. In the delta band, we observed similar phase angles for both groups (F(1,62) = 0.494, *p* < 0.470), indicating comparable phase locking at this frequency, with small angles indicating the absence of delta band phase-shift between modalities. Yet, a significant group effect was observed in the theta band. In children with ASD the phase-shift amounted to 180 degrees and the group difference was significant (F(1,62) = 12.05, *p* < 0.001) (Fig. 6). The observed 180-degree phase shift in autism could suggest that auditory and visual information is out of sync: when one sensory modality is at its peak processing efficiency, the other is at its lowest, potentially leading to disjointed, even conflicting sensory processes.

### AV phase-shift is related to auditory encoding accuracy in TD and visual encoding accuracy in autism

**Fig. 7:**
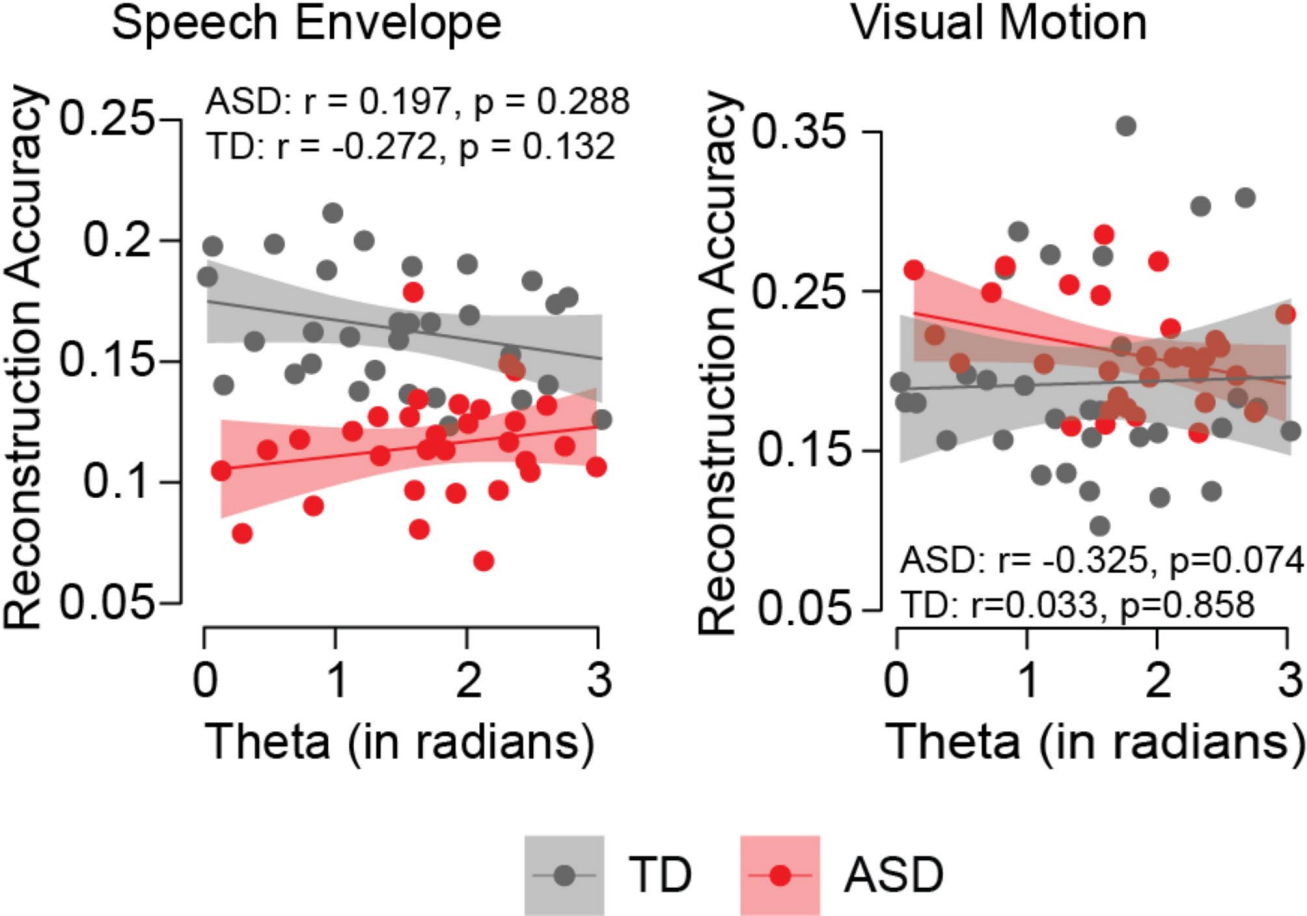
The relationship between theta phase-shift and reconstruction accuracy of speech envelope (left) and visual motion (right) in ASD and TD. ASD group suggests a greater phase shift between speech envelope and visual motion positively correlates with speech reconstruction accuracy but negatively correlates with visual reconstruction, while reversely in TD group

Finally, we explored the relationship between the AV phase-shift in the theta band and the accuracy of auditory and visual information reconstruction within an unimodal framework (Fig. 7). As expected, in TD children the AV phase-shift did not influence visual reconstruction accuracy (*r* = 0.033, *p* = 0.858), but there was a weak negative correlation between the phase-shift extent and speech reconstruction accuracy (*r* = -0.272, *p* = 0.132): when the AV phase-shift increased speech reconstruction accuracy decreased, which given the visual lead previously observed could suggest a causal effect. A different pattern was seen in children with ASD, with no relation between the phase shift extent and speech reconstruction accuracy (*r* = 0.197, *p* = 0.288; group*phase-shift t = -1.835, *p* = 0.072), but a weak negative correlation between the phase-shift extent and the accuracy of visual information reconstruction (*r* = -0.325, *p* = 0.074; group*phase-shift t = 1.062, *p* = 0.293), with larger AV phase-shifts linked to poorer visual reconstruction accuracy. Similarly, given the auditory lead observed in the group with ASD, this could suggest a causal effect.

## Discussion

Using several analyses of the EEG recorded in very young children with and without ASD while they were watching a short animated movie, we confirmed previous results showing profound anomalies in the capacity to follow speech rhythms [24, 89], an essential prerequisite to speech comprehension. The present study goes beyond this observation by showing that children with ASD did not exhibit the natural dominance of auditory processing when exposed to natural audio-visual speech conditions. Instead, audio-visual processing was impacted by a temporal misalignment of these sensory inputs, which disrupted the predictive processing typically at play when perceiving speech.

### Audio-visual integration anomalies interfere with sensory encoding in ASD

The synchronization of the two sensory modalities plays a pivotal role in understanding the communicative challenges observed in ASD. Our study reveals that speech processing anomalies in ASD from an early developmental stage [19–24] are not merely isolated auditory deficits but are deeply connected to the integration of auditory and visual information, a process critical for effective communication, particularly in dynamic or complex listening environments [90, 91].

Our findings reveal a specific disruption in audio-visual integration among children with ASD, manifesting in visual dominance and temporal disorganization in auditory and visual processing. This disruption sharply contrasts with the expected auditory processing dominance [92] and might significantly contribute to the language development difficulties encountered by these children. In TD, the precedence of orofacial visual cues during speech facilitates auditory comprehension through predictive processing, optimizing the brain’s synchronization to incoming speech signals [93]. In ASD, extended integration time windows and the lack of effective synchronization of auditory responses by visual signals (as evidenced by the atypical theta band phase-shifts) suggest they do not use visual cues to facilitate auditory speech processing, and that on the contrary auditory cues disrupt the visual processing in communicative situations.

### Repercussions of disrupted audio-visual processing on speech tracking

Children with ASD exhibit visual motion tracking and processing capabilities comparable to their TD peers despite different scene analysis patterns, as previously observed [13, 94]. Within their preferred exploration zones, children with ASD process visual motion similarly to TD children [94] with an equivalent level of bottom-up excitability to visual stimuli [95, 96]. The univariate encoding results indeed suggest that the neural activity responsible for visual motion tracking operates similarly in both ASD and TD groups.

However, when visual processing co-occurs with speech processing, difficulties appear. Our multivariate modeling indicates that the neural encoding of audio-visual percepts in ASD children is less efficient, confirming that audiovisual contexts can disrupt brain responses to speech in this population [97]. Our study reinforces this crucial finding by showing that while autistic children encode single visual streams relatively well (visual motion tracking in univariate model), they struggle to concurrently encode auditory and visual streams (multivariate model).

Our study uncovers the potential underpinnings of the audiovisual integration difficulties observed in autism. The decoding results further indicate that while audio-visual integration interferes with visual processing in both groups, and that its impact on speech processing is particularly detrimental in the ASD group. Thus, the impairments in AV integration we observe are not merely additive but exacerbate sensory processing challenges in ASD. This framework explains that even though 12-month-old infants at risk for ASD explore faces and mouths similarly to infants with no family history of autism [98], they cannot leverage audiovisual cues for language acquisition as do typical children.

### Audio-visual temporal integration underlies speech impairment in autism

Audiovisual integration relies on the temporal alignment of sensory events, with visual cues enhancing auditory clarity, especially in noisy or ambiguous conditions [99–101]. Our findings confirm in TD children a visual lead (~ 50 ms) within a temporal window that is conducive to effective interaction and coordination between auditory and visual cues [102]. This window reasonably aligns with established models, positing a 200 ms integration period [39, 61–63], ranging from a 30 ms visual lag to a 170 ms of visual lead [61].

The precise timing of auditory and visual sequences is fundamental to audio-visual integration via predictive processing, whereby the brain leverages visual cues to anticipate and decode forthcoming auditory information. Here, phase-locking analyses in TD children show that the neural responses associated with auditory and visual processing exhibit a 90-degree phase shift, indicating that such a phase relationship optimizes a dynamic balance between the sensory streams, facilitating integration and enhancing perception and communication [101, 103]. The pivotal role of the theta frequency band in orchestrating audio-visual speech processing is robustly supported in the literature [56, 58, 59]. A pi/2 visual lead results in aligning visual information processing with the auditory inputs. This reliable phase alignment observed in TD children sharply contrasts with the broad phase distribution observed in the ASD group, signalling inconsistent audio-visual integration. In logic, reconstruction accuracy is a proxy of sensory encoding accuracy. Thus, in TD children, the relationship between phase-shift and reconstruction accuracy confirms the known reliance on visual cues to enhance auditory processing, with any misalignments adversely affecting speech information integration. Conversely, in ASD children, while speech encoding is weaker and overall less dependent on visual-auditory phase congruency, visual processing is vulnerable to strong AV resynchronization.

The atypical auditory lead (~ 50 ms) observed in ASD indicates that audio-visual integration is jeopardized and that the conventional sequence where visual information typically precedes auditory is inverted. Furthermore, the180-degree phase-shift in the neural activities associated with each stream reflects a profound disruption in temporal coordination, potentially leading to confusion or interpretation errors. Such a discrepancy underscores a critical deficiency in predictive processing in ASD, where, rather than synergistically enhancing each other, auditory and visual cues conflict, undermining the synthesis of coherent audio-visual perception [31, 32].

Our results are consistent with the notion that the phase of low-frequency neural oscillations is crucial for the temporal parsing in speech [104]. The anomaly in temporal encoding mechanisms described in our experiment is constrained by the temporal features provided by external stimulation to build a temporal reference frame. While delta oscillations have previously been linked to temporal predictability [102, 104], we observed here that sensory integration is affected by AV misalignment in the theta range, which is associated with atypical speech perception in ASD. AV integration primarily occurs at the syllable level with a typical tolerance to AV asynchrony around 250ms, which corresponds to the theta range [39, 61–64].

## Conclusion

Our results show markedanomalies in audio-visual integration in young children with ASD that provide specific underpinnings for previous findings depicting disrupted speech rhythm tracking. They further reveal that disruption in audio-visual integration, manifesting as temporal desynchronization, impacts speech processing and contributes to the communicative challenges in autism. Our results also highlight the critical role of temporal processing in audio-visual integration and underscore the importance of characterizing these mechanisms in ASD. Moving forward, these insights could inform the development of targeted interventions aimed at regulating temporal speech processing and AV synchronization to improve communication in ASD children.

## Electronic supplementary material

Below is the link to the electronic supplementary material.

Supplementary Material 1

Supplementary Material 2

## Data Availability

The unprocessed datasets for this manuscript are not publicly available yet due to ongoing analysis as part of a longitudinal study. The results are expected to be published in the future. Once all data has been published, requests to access the datasets should be directed to Dr. Marie Schaer at marie.schaer@unige.ch. The custom MATLAB analysis scripts will be made available upon request to the xiaoyue.wang@pasteur.fr.

## Declarations

### Preprint servers

The manuscript was deposited as a preprint in bioRxiv with the license CC BY-NC-ND 4.0.
