# Supplementary material for: Atypical audio-visual neural synchrony and speech processing in early autism
Source: J Neurodev Disord. 2025 Feb 18;17:9. doi: 10.1186/s11689-025-09593-w (PMC11837391; doi:10.1186/s11689-025-09593-w)
Supplement: Supplementary file 1 — Supplementary Material 1 [file 11689_2025_9593_MOESM1_ESM.docx]

| Supplementary Table 1 EEG data quality checkable table | | | | | |
| --- | --- | --- | --- | --- | --- |
| Trial # | | Trial Length (ms) | | Channel # | |
| TD | ASD | TD | ASD | TD | ASD |
| 36 | 34 | 2338.5278 | 2476.9706 | 11 | 3 |
| 36 | 36 | 2338.3889 | 2489.9167 | 15 | 6 |
| 36 | 36 | 2404.6944 | 2489.9167 | 13 | 8 |
| 35 | 36 | 2378.5714 | 2489.9167 | 13 | 7 |
| 36 | 36 | 2404.1111 | 2489.9167 | 11 | 13 |
| 33 | 35 | 2518.7273 | 2334.6857 | 18 | 5 |
| 33 | 33 | 2175.7576 | 2372.9091 | 6 | 9 |
| 35 | 25 | 2501.1429 | 2346.96 | 3 | 14 |
| 36 | 36 | 2374.6667 | 2401.3611 | 4 | 13 |
| 36 | 22 | 2460.7778 | 2602.4545 | 3 | 16 |
| 36 | 35 | 2489.9167 | 2523.4571 | 4 | 17 |
| 36 | 32 | 2401.3611 | 2369.4375 | 6 | 12 |
| 36 | 36 | 2489.9167 | 2489.9167 | 2 | 27 |
|  | 35 |  | 2524.8286 |  | 4 |
| 36 | 27 | 2489.9167 | 2298.6296 | 6 | 6 |
| 36 | 28 | 2489.9167 | 2695.6071 | 3 | 6 |
| 36 | 36 | 2489.9167 | 2489.9167 | 6 | 9 |
| 31 | 35 | 2384.1613 | 2434.0857 | 9 | 3 |
| 36 | 36 | 2489.9167 | 2489.9167 | 5 | 2 |
| 36 | 35 | 2489.9167 | 2403.4 | 8 | 4 |
| 36 | 35 | 2489.9167 | 2428.7429 | 5 | 7 |
| 31 | 35 | 2370.7742 | 2498.5714 | 12 | 3 |
| 36 | 25 | 2489.9167 | 2288.08 | 10 | 6 |
| 35 | 36 | 2489.9167 | 2489.9167 | 10 | 8 |
| 36 | 36 | 2489.9167 | 2489.9167 | 7 | 4 |
| 36 | 36 | 2489.9167 | 2489.9167 | 6 | 3 |
| 28 | 35 | 2125.6429 | 2301.5143 | 3 | 8 |
| 36 | 36 | 2374.8333 | 2489.9167 | 16 | 11 |
| 35 | 32 | 2518.1429 | 2343.625 | 4 | 12 |
| 36 | 21 | 2374.1111 | 2476.6667 | 5 | 10 |
| 36 | 31 | 2489.9167 | 2574 | 5 | 6 |
| 35 | 33 | 2534.9429 |  | 5 |  |
| 35 |  | 2489.9167 |  | 11 |  |
| *Trial #: kept trial number after rejection* | | | | | |
| *Trial length: averaged length of kept trial* | | | | | |
| *Channel #: interpolated channel number* | | | | | |
|  |  |  |  |  |  |
|  |  |  |  |  |  |
|  |  |  |  |  |  |
|  |  |  |  |  |  |
