# Supplementary material for: Atypical audio-visual neural synchrony and speech processing in early autism
Source: J Neurodev Disord. 2025 Feb 18;17:9. doi: 10.1186/s11689-025-09593-w (PMC11837391; doi:10.1186/s11689-025-09593-w)
Supplement: Supplementary file 2 — Supplementary Material 2 [file 11689_2025_9593_MOESM2_ESM.docx]

Supplementary Materials

Methods

**Selection of optimal regularization parameter**

To mitigate the risk of data overfitting in forward encoding models, we integrated an optimized regularization parameter λ, which weights the diagonal of the autovariance matrix before inversion in Tikhonov regularization or ridge regression [[85]](https://www.zotero.org/google-docs/?dVEidE), determined for each stimulus feature. To achieve this, we trained multiple model iterations on subsets of data comprising n-1 participants. During these iterations, λ was systematically adjusted within a predefined range from 10^0^ to 10^5^, with increments in the exponent of 0.5. The criterion for selecting the optimal λ was maximal predictive accuracy. This was quantitatively assessed by calculating Pearson's correlation coefficient (r) between the predicted EEG signals and the observed signals, for each electrode and each participant. Once optimal λ was identified, the refined model was used to predict the EEG responses of the nth participant, using an n-fold leave-one-out cross-validation paradigm. This methodology was applied consistently across both autism and TD groups, ensuring the robustness and reliability of the predictive models within each group.

*
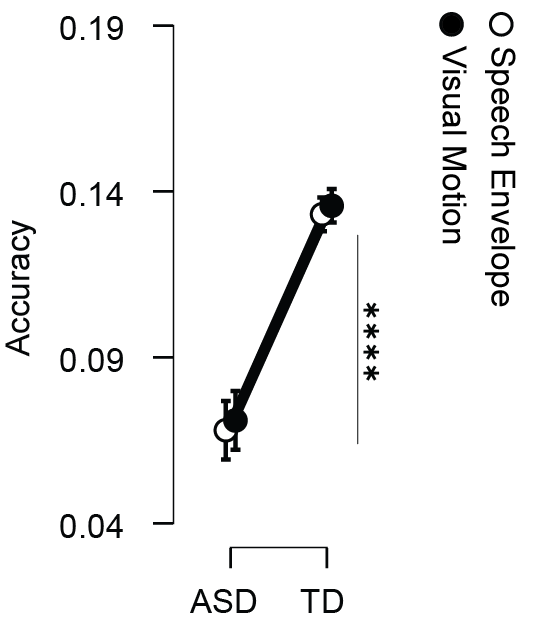
*

*Supplement Figure 3-1 ASD and TD groups' decoding accuracy in the AV-joint model. This figure presents the reconstruction accuracies for stimuli, specifically the speech envelope and visual motion from AV joint model. The error bars represent the standard error of the mean. Significance levels are indicated as follows:‘ns’ for p>0.05 (not significant), * for p <0.05, ** for p<0.01, *** for p<0.001, **** for p<0.0001.*
